# Role of Na^+^, K^+^, Cl^−^, proline and sucrose concentrations in determining salinity tolerance and their correlation with the expression of multiple genes in tomato

**DOI:** 10.1093/aobpla/plu039

**Published:** 2014-07-04

**Authors:** Pedro Almeida, Richard Feron, Gert-Jan de Boer, Albertus H. de Boer

**Affiliations:** 1 Faculty of Earth and Life Sciences, Department of Structural Biology, Vrije Universiteit Amsterdam, NL-1081 HV Amsterdam, The Netherlands; 2 Enza Zaden, Research and Development, Haling 1/E, 1602 DB Enkhuizen, The Netherlands

**Keywords:** Genetic and physiological parameters, salinity stress, tomato accessions.

## Abstract

One of the major abiotic stresses affecting agriculture is soil salinity, which reduces crop yield and, consequently, revenue for farmers. Although tomato is an important agricultural species, elite varieties are poor at withstanding salinity stress. Thus, a feasible way of improving yield under conditions of salinity stress is to breed for improved salt tolerance. In this study, we analysed the physiological and genetic parameters of 23 tomato accessions in order to identify possible traits to be used by plant breeders to develop more tolerant tomato varieties. Although we observed a wide range of Na^+^ concentrations within the leaves, stems and roots, the maintenance of growth in the presence of 100 mM NaCl did not correlate with the exclusion or accumulation of Na^+^. Nor could we correlate the growth with accumulation of sugars and proline or with the expression of any gene involved in the homoeostasis of Na^+^ in the plant. However, several significant correlations between gene expression and Na^+^ accumulation were observed. For instance, Na^+^ concentrations both in the leaves and stems were positively correlated with *HKT1;2* expression in the roots, and Na^+^ concentration measured in the roots was positively correlated with *HKT1;1* expression also in the roots. Higher and lower Na^+^ accumulation in the roots and leaves were significantly correlated with higher *NHX3* and *NHX1* expression in the roots, respectively. These results suggest that, in tomato, for a particular level of tolerance to salinity, a complex relationship between Na^+^ concentration in the cells and tissue tolerance defines the salinity tolerance of individual tomato accessions. In tomato it is likely that tissue and salinity tolerance work independently, making tolerance to salinity depend on their relative effects rather than on one of these mechanisms alone.

## Abstract (precis)

One of the major abiotic stresses affecting agriculture is soil salinity, which reduces crop yield. A way of improving yield in conditions of salinity stress is to breed for improved salt tolerance. In this study, we analysed physiological and genetic parameters of 23 tomato accessions in order to identify possible traits to be used by plant breeders to develop more tolerant tomato varieties. Our results suggest that, in tomato, for a particular level of tolerance to salinity, a complex relationship between Na^+^ concentration in the cells and tissue tolerance defines the salinity tolerance of individual tomato accessions.

## Introduction

One of the major abiotic stresses that agriculture faces is salinity (Munns and Tester 2008; Food and Agriculture Organization (FAO) 2009). An excessive amount of salt in the soil negatively affects plant germination, growth, development and productivity (Manaa *et al.* 2011). A very important mechanism of salinity tolerance is the ability to minimize the amount of Na^+^ entering the plant via the roots (Laurie *et al.* 2002; Tester and Davenport 2003; Munns and Tester 2008). The control of Na^+^ transport at the cellular level by secreting and sequestering it in tissues, cells or organelles, where Na^+^ causes less harm, is also critical to the achievement of salinity tolerance (Munns and James 2003; Parida and Das 2005). In fact, a great contributor to salinity stress is the accumulation of high concentrations of Na^+^ in the leaf cell cytoplasm (Jha *et al.* 2010).

In some species, however, Cl^−^ is the main stressful ion (Prior *et al.* 2007). This does not mean that Cl^−^ has a higher metabolic toxicity than Na^+^, but that these species are better at excluding Na^+^ than Cl^−^ (Munns and Tester 2008). Interestingly, several studies show that, besides Na^+^ exclusion, tissue tolerance to high Na^+^ concentrations also plays an important role in salinity tolerance (El-Hendawy *et al.* 2004; Genc *et al.* 2007; Jha *et al.* 2010). For instance, it was shown that in wheat and *Arabidopsis* no clear relationship between Na^+^ exclusion and salinity tolerance exists (Rus *et al.* 2006; Genc *et al.* 2010; Jha *et al.* 2010). In fact, two *Arabidopsis thaliana* ecotypes—Tsu1 and Ts—accumulated more Na^+^ in the shoots than several other *Arabidopsis* ecotypes studied (Rus *et al.* 2006). Nevertheless, the Tsu1 and Ts ecotypes were more salt tolerant than those accumulating less Na^+^ in the shoots. The understanding of the mechanisms involved in the uptake and movement of Na^+^ throughout the plant as well as the genes involved in Na^+^ homoeostasis *in planta* is crucial (Jha *et al.* 2010) in the improvement of the salinity tolerance of current crop varieties.

Several genes have been shown to play a role in the control of Na^+^ movement throughout the plant. For example, *HKT* (Berthomieu *et al.* 2003; Ren *et al.* 2005; Davenport *et al.* 2007; Moller *et al.* 2009; Jha *et al.* 2010; Ali *et al.* 2012), *SOS* (Qiu *et al.* 2002; Shi *et al.* 2002), *NHX* (Gaxiola *et al.* 1999), *AVP* (Gaxiola *et al.* 2001; Zhao *et al.* 2006) and *AHA* (Apse *et al.* 1999) gene families encompass such genes. Studies carried out with *Arabidopsis* (Uozumi *et al.* 2000; Berthomieu *et al.* 2003; Davenport *et al.* 2007; Moller *et al.* 2009), rice (Ren *et al.* 2005; Jabnoune *et al.* 2009; Yao *et al.* 2010), *Eucalyptus* (Fairbairn *et al.* 2000), barley (Mian *et al.* 2011) and *Thellungiella* (Ali *et al.* 2012) show that *HKT* genes are involved in the control of the Na^+^ movement throughout the plant. The *HKT* gene family encodes proteins that are responsible for the influx of Na^+^ into cells (Uozumi *et al.* 2000; Moller *et al.* 2009). This family is divided into two subfamilies depending on the nucleic acid sequences and protein structure of their members (Platten *et al.* 2006). Members of Subfamily 1 have an important role in salinity tolerance (Uozumi *et al.* 2000; Ren *et al.* 2005). *Athkt1;1* mutants and the *Arabidopsis* ecotypes Tsu1 and Ts1 (Rus *et al.* 2006) have no *AtHKT1;1* expression in the roots. This is related with hyperaccumulation of Na^+^ in the shoots and reduced accumulation in the roots (Rus *et al.* 2006). Cell-type specific over-expression of *AtHKT1;1* in the root stele of *Arabidopsis* plants (Moller *et al.* 2009), and in the root cortex of rice plants (Plett *et al.* 2010), results in a significant decrease of Na^+^ accumulation in the shoots and in the increase of tolerance to salinity in these plants.

The plasma membrane Na^+^/H^+^ antiporters SOS1 family encompasses members that have been implicated in reducing the quantity of Na^+^ translocated to the shoots via the transpiration stream (Qiu *et al.* 2002; Shi *et al.* 2002). *AtSOS1* is expressed in the epidermal cells of the root tissue (Shabala and Cuin 2008), where it pumps Na^+^ into the external medium, and it is expressed in cells along the vascular tissue (Shi *et al.* 2000), where it pumps Na^+^ into the transpiration stream (Shi *et al.* 2000, 2002). Shi *et al.* (2003) show that over-expression of *AtSOS1* in *Arabidopsis* improves its salt tolerance through the limitation of Na^+^ accumulation in plant cells.

Another ion transport protein, with a very important role in salinity tolerance, is the vacuolar Na^+^, K^+^/H^+^ antiporter AtNHX1 (Venema *et al.* 2003). This transporter is responsible for the detoxification of the cytoplasm by pumping Na^+^ into the vacuole (Gaxiola *et al.* 1999). Several studies show that over-expression of *AtNHX* or its homologues from other species improves the salt tolerance of plants (Apse *et al.* 1999; Zhang and Blumwald 2001; Xue *et al.* 2004; He *et al.* 2005; Chen *et al.* 2007). Many transport processes that occur in plants are directly or indirectly energized by the proton gradient across membranes produced by H^+^-pumping ATPases (Maathuis *et al.* 2003). At the plasma membrane, the *AHA* gene family encodes P-type ATPases that create an H^+^ gradient used to energize, among other processes, the extrusion of Na^+^ (Apse *et al.* 1999) via SOS1. High *AHA* expression levels are always observed in cells where intense active transport takes place (Palmgren and Harper 1999; Palmgren and Nissen 2011). One of the causes for intense active transport is the excess of Na^+^ ions entering the root. *AtAVP1* encodes a vacuolar H^+^-translocating pyrophosphatase (H^+^-PPase) that transports H^+^ across the tonoplast (Munns and James 2003). This gradient results in the energization of the movement of Na^+^ into the vacuoles through Na^+^, K^+^/H^+^ antiporters like AtNHX1 (Gaxiola *et al.* 2001). When *AtAVP1* is over-expressed in *Arabidopsis*, an increase in salinity tolerance has been observed caused by a better sequestration of Na^+^ in the vacuole (Gaxiola *et al.* 2001).

In plants suffering from salinity stress, the production of compatible solutes has also shown to be an effective mechanism to protect plants. Both amino acids and sugars are non-toxic compounds that accumulate preferentially in the cytoplasm, helping not only to maintain the turgor and osmotic balance but also to protect the cell structure (Conde *et al.* 2011). Salinity stress causes the accumulation of proline in several plant species including tomato (Fujita *et al.* 1998). Proline functions both as an osmolyte and as an osmoprotectant (Conde *et al.* 2011). It can be synthesized via two different pathways from either glutamate or ornithine (Hmida-Sayari *et al.* 2005); however, the glutamate pathway seems to be the predominant pathway in conditions of osmotic stress (Silva-Ortega *et al.* 2008). The first two steps of proline biosynthesis from glutamate are catalysed by the enzyme Δ^1^-pyrroline-5-carboxylate synthetase (*P5CS*) (Hu *et al.* 1992). The *P5CS* gene was isolated in several species, such as rice (Yoshiba *et al.* 1995), *Arabidopsis* (Igarashi *et al.* 1997) and tomato (Fujita *et al.* 1998). Its induction and the accumulation of proline correlated in rice and *Arabidopsis* (Yoshiba *et al.* 1995; Igarashi *et al.* 1997). Several studies showed that salt-tolerant plants had higher accumulation of proline in response to salinity treatments than salt-sensitive ones (Jain *et al.* 1991; Kirti *et al.* 1991). However, some studies showed the opposite. Working with tomato, Aziz *et al.* (1998) reported a negative correlation between salt tolerance and proline accumulation. Similar results were reported by Tal *et al.* (1979).

Salinity stress also causes the accumulation of sucrose (Juan *et al.* 2005). Sucrose is not toxic in the cytoplasm where it allows the maintenance of turgor and protects the structure of molecules against the deleterious effects of water scarcity (Popp and Smirnoff 1995; Murakeozy *et al.* 2003; Juan *et al.* 2005).

Tomato is one of the most important horticultural crops (Villalta *et al.* 2008). Unfortunately, elite varieties can withstand salinity stress poorly. This is a result of the usual breeding strategies, which have as target yield increments under optimal conditions (Villalta *et al.* 2008). Because of this, only 10 % of all genetic variability among all tomato species is present in elite tomato varieties (Miller and Tanksley 1990). The enormous genetic variation present in wild tomato species can be very important in the development of new salinity-tolerant cultivars. The introduction of genes from wild species of tomato can be achieved by crossing *S. lycopersicum* with other *Solanum* species of the *S. lycopersicum* complex: *Solanum pimpinellifolium*, *S. neorickii*, *S. chmielewskii* and *S. pennellii*. Tolerance means adaptation and wild *Solanum* species are adapted to marginal environments. For this reason, modern breeding programmes can take advantage of adaptations present in wild *Solanum* species (Villalta *et al.* 2008). These adaptations in tomato are studied independently in a number of other papers (Tal *et al.* 1979; Bolarin *et al.* 1995; Aziz *et al.* 1998; Fujita *et al.* 1998; Juan *et al.* 2005; Villalta *et al.* 2008; Galvez *et al.* 2012; Huertas *et al.* 2012); however, to our knowledge our study is the first to investigate a high number of salt-tolerance-associated traits in one single experiment.

The present study tried to define these adaptations to salt tolerance in 23 tomato accessions. We evaluated the role of Na^+^, K^+^, Cl^−^, proline and sucrose concentrations in determining salinity tolerance, and correlated these traits with the expression of genes involved in Na^+^ homoeostasis.

## Methods

### Plant material

Seeds of 23 different tomato accessions: *Solanum chilense* LA 1938 and LA 1959; *S. chmielewskii* LA 1325 and LA 2695; *S. corneliomuelleri* GI 568 and PI 126443; *S. galapagense* LA 0532 and LA 0317; *S. habrochaites* G1560 and LA 2167; *S. habrochaites glabratum* LA 2860 and PI 126449; *S. lycopersicum* Abigail F1, LA 3320, LA 2711 and Arbasson F1; *S. neorickii* LA 2194; *S. pennellii* LA 1340 and LA 1522; *S. pennellii puberulum* LA 1302; *S. peruvianum* LA 2548; and *S. pimpinellifolium* OT 2209 and LA 1245 were surface sterilized by soaking in 1 % (V/V) commercial sodium hypochlorite solution for 15 min and rinsing with sterile water three times. After sterilization, seeds were sown in rock wool plugs soaked with half-strength Hoagland's solution (one seed per rock wool plug). Plugs were covered with dry vermiculite to avoid dehydration. We used a randomized design consisting of two NaCl treatments with three biological replicas for each of the 23 accessions. Each biological replica consisted of a pool of 7–10 plants. On alternate days, plants were irrigated with half-strength Hoagland's solution. Plants were kept in a climate chamber under a 14/10 hours photoperiod and a 20/18 °C day/night temperature. Salt treatment started 2 weeks after sowing. Salt-treated plants were irrigated with half-strength Hoagland's solution supplemented with 50 mM NaCl, the excess solution was allowed to drain. Two days later, salt-treated plants were irrigated with half-strength Hoagland's solution supplemented with 100 mM NaCl. Control plants were irrigated with half-strength Hoagland's solution only. Plants were irrigated every 2 days over 3 weeks. Plants were harvested after 3 weeks of treatment. Root, stem and leaf tissue of each biological replica of each accession was harvested in tubes, snap-frozen in liquid nitrogen and transferred to an ultra-freezer, where they were stored at −80 °C. Before snap-freezing root samples were rinsed with demineralized water to remove Na^+^ from the medium present on the roots. Frozen samples were dried using a freeze dryer (Christ Alpha 1-4 LD plus, Germany) for 1 week. When completely dry, samples were ground into a fine powder and stored in closed tubes at room temperature.

### Na^+^, K^+^ and Cl^−^ measurements

For the quantification of Na^+^ and K^+^, between 50 and 100 mg of dried material was weighed in 2 mL tubes. One millilitre of ultrapure water (Fluka Analytical, Sigma-Aldrich, USA) was added and the tubes were boiled for 10 min at 100 °C. Samples were then filtered in a 96-wells filter plate (Thermo Scientific, Rochester, NY, USA) through centrifugation at 3000 rpm for 3 min. For Na^+^ and K^+^ measurements, 6 µL of the filtrate was diluted in 6 mL of ultrapure water (Fluka Analytical, Sigma-Aldrich), and the resulting solution was analysed for Na^+^ and K^+^ concentrations using an atomic absorption spectrometer (AAnalyst 200; PerkinElmer AAS). The AAS was calibrated using sodium and potassium atomic spectroscopy standard concentrate (Fluka Analytic, Sigma-Aldrich), and the average of three technical replicas was used for the ion concentration calculations. Cl^−^ ions were measured directly on the filtered samples, also used for Na^+^ and K^+^ measurements, on a chloride counter (MKII Chloride Analyzer 926; Sheerwood, UK). The chloride counter was calibrated with 500 μL of Chloride Meter Standard (Sheerwood).

### Sucrose and proline measurements

Ten milligrams of dried leaf material was weighed and inserted in 2 mL tubes. One millilitre of 80 % ethanol was added to each sample and the tubes were incubated for 90 min at 70 °C in a water bath. Samples were regularly vortexed to assure an efficient ethanol extraction of the sugars. After incubation, samples were centrifuged at full speed (14 000 RPM) for 10 min at room temperature. About 800 μL of the supernatant was transferred into a new 2 mL tube and 800 μL of Milli-Q water was added. Ten microlitres of this solution was used with the Sucrose/D-Glucose/D-Fructose-UV test Kit (R-Biopharm, Germany) to quantify sugars according to the manufacturer's protocol. For the calibration, 250 mg of glucose, sucrose and fructose were dissolved in a 250 mL volumetric flask containing 40 % ethanol. A dilution series was made (0, 2, 4, 8, 16 times dilution) and used for the standard curve. For the quantification of proline, between 20 and 30 mg dry leaf material from control and salt-treated plants were used. Extraction and quantification were made according to the method of Bates *et al.* (1973).

### RNA extraction, complementary DNA synthesis and quantitative PCR

For the extraction of RNA, 30 mg of frozen material (root and leaf) was used. A NucleoSpin 96 RNA Kit (Macherey-Nagel) was used and RNA was extracted according to the manufacturer's protocol. After extraction, 5 μL of RNA was incubated at 37 °C for 10 min and tested in an agarose gel to check for the quality. The concentration was measured using a NanoDrop (ThermoScientific). First-strand cDNA was synthesized using 1 μg of total RNA, 200 Units of SuperScript II Reverse Transcriptase (Invitrogen Life Technologies), 1 mM dNTPs, 100 mM DTT, 5× first-strand buffer and 10 μM oligo dT primer, at 37 °C for 50 min. cDNA was used as a template for quantitative real-time PCR (qRT–PCR). Expression values were calculated according to the $C^{-\Delta \Delta C_{t}}$ method of Livak and Schmittgen (2001) with *SlElfα* as an internal reference. Efficiencies between 95 and 105 % were regarded as acceptable. Amplification efficiencies were checked to ensure they were within the same range as the target genes. Cycle thresholds (*C*_t_) values were determined in the automatic mode. Only slope values with an *R*^2^ > 0.990 were taken into account. The specificities of all primers were checked by running a melting curve. Primers used in this experiment can be seen in **Supporting Information**.

### Statistical analysis

To assess the effect of salt treatment on Na^+^, K^+^, Na^+^/K^+^ and Cl^−^ levels per accession, we used the Student's *t*-test. If the values were not normally distributed or if the assumption of homogeneity of variance was violated, we transformed the data. Gene expression data were transformed either logarithmically or ranked transformed. We performed Pearson's correlations between the different accessions or in the case of rank transformed data, we performed Spearman's rank correlations. All analyses were conducted using SPSS 17.0.

## Results

### Plant tolerance index and Na^+^, proline and sucrose concentrations

Almost all salt-stressed plants showed a significant reduction in shoot fresh weight (FW) over the 2-week period of salt treatment **[see** **Supporting Information****]**. Accessions GI 568, LA 1325, PI 126443, LA 1245, Abigail, LA 1340 and LA 1522 showed no significant reduction in shoot FW when treated with NaCl. With the exception of Abigail F1, accessions without a significant reduction in shoot FW showed the highest scores according to the plant tolerance index (PTI) (see Jha *et al.* 2010) **[see** **Supporting Information****]**. In this study, we defined PTI as the ratio between the FW of salt-treated shoots divided by FW of control shoots. We did not use total plant FW, because it was not possible to harvest the total amount of roots produced by each individual plant. Based on the PTI, the most salt-sensitive accession was LA 1938 (PTI = 0.37) and the most salt-tolerant accessions was LA 1245 (PTI = 0.98).

**Figure 1.**
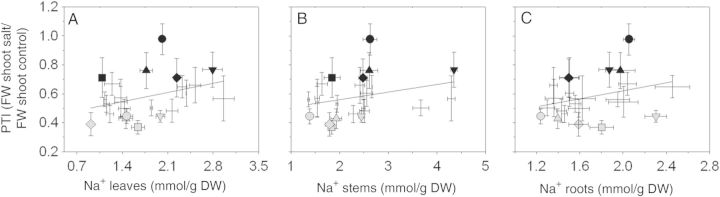
Relationship between PTI and Na^+^ accumulation in the (A) leaves, (B) stems and (C) roots of tomato plants treated with 100 mM NaCl for 2 weeks before being harvested. Black symbols represent the five accessions with higher PTI values: LA 1325 (square), PI 126443 (rhombus), LA 1522 (up-pointing triangle), LA 1340 (down-pointing triangle) and LA 1245 (filled circle). Grey symbols represent the five accessions with lower PTI values: LA 1938 (square), LA 3320 (rhombus), LA 2711 (up-pointing triangle), LA 2548 (down-pointing triangle) and OT 2209 (filled circle). Values indicate the means ± SE of three biological replicas. No statistically significant correlation was found for any of the three correlations. PTI vs. [Na^+^] leaves and PTI vs. [Na^+^] roots were tested using the Pearson correlation coefficient, whereas PTI vs. [Na^+^] stems was tested using the Spearman correlation coefficient.

Variation could also be observed in the concentrations of Na^+^, K^+^ and Cl^−^ and the Na^+^/K^+^ ratio measured in the three tissues analysed of all accessions **[see** **Supporting Information****]**. All accessions showed significant increases in Na^+^ and Cl^−^ concentrations and Na^+^/K^+^ ratios, in all tissues, when treated with 100 mM NaCl. Interestingly, these results showed no correlation with the PTI values (Fig. 1). In salt-treated plants, not all accessions showed a significant reduction in K^+^ concentrations.

Proline and sucrose concentrations of both control and salt-treated plants were measured in the leaves. All tomato plants treated with 100 mM NaCl for 3 weeks showed a significant increase in the concentration of both proline and sucrose (data not shown). In salt-treated plants, no correlation between proline or sucrose concentrations and PTI scores was observed (data not shown).

### *P5CS* expression, proline and sucrose content in the leaves

**Figure 2.**
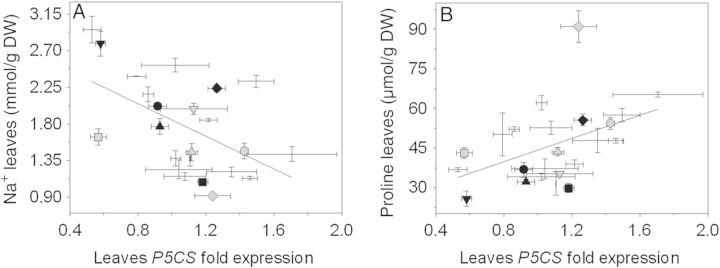
Relationship between (A) Na^+^ concentration and (B) proline concentration and *P5CS* gene expression in the leaves. Black symbols represent the five accessions with higher PTI values: LA 1325 (square), PI 126443 (rhombus), LA 1522 (up-pointing triangle), LA 1340 (down-pointing triangle) and LA 1245 (filled circle). Grey symbols represent the five accessions with lower PTI values: LA 1938 (square), LA 3320 (rhombus), LA 2711 (up-pointing triangle), LA 2548 (down-pointing triangle) and OT 2209 (filled circle). Values indicate the means ± SE of three biological replicas. Both correlations tested using the Pearson correlation coefficient. Both correlations are statistically significant.

We analysed the *P5CS* gene expression and compared it to both Na^+^ (Fig. 2A) and proline (Fig. 2B) accumulation in the leaves. In the leaves there was a clear link between *P5CS* gene expression, accumulation of proline and accumulation of Na^+^. The higher the *P5CS* expression, the higher the accumulation of proline, and the lower the accumulation of Na^+^. This correlation, however, was not observed in the roots (data not shown).

### Gene expression analysis of transporters involved in Na^+^ homoeostasis, Na^+^ accumulation, and *NHXs* and *AVPs* expression

**Figure 3.**
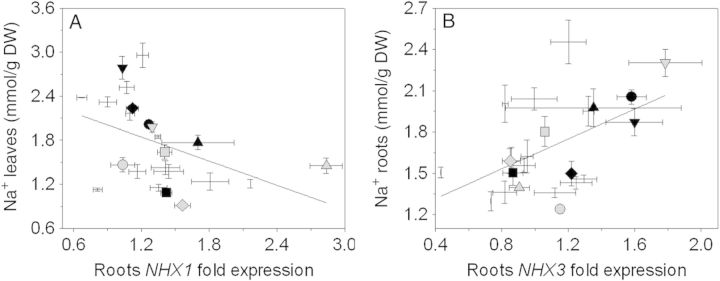
Correlations between (A) Na^+^ accumulation in the leaves and *NHX1* expression in the roots and (B) Na^+^ accumulation in the roots and *NHX3* expression in the roots. Black symbols represent the five accessions with higher PTI values: LA 1325 (square), PI 126443 (rhombus), LA 1522 (up-pointing triangle), LA 1340 (down-pointing triangle) and LA 1245 (filled circle). Grey symbols represent the five accessions with lower PTI values: LA 1938 (square), LA 3320 (rhombus), LA 2711 (up-pointing triangle), LA 2548 (down-pointing triangle) and OT 2209 (filled circle). Values indicate the means ± SE of three biological replicas. Values indicate the means ± SE of three biological replicas. Correlations (A) tested using the Spearman correlation coefficient. Correlations (B) tested using the Pearson correlation coefficient. Both correlations are statistically significant.

Salt-treated accessions showed a correlation between Na^+^ accumulation and *NHX* expression (Fig. 3). Lower Na^+^ accumulation in the leaves was correlated with higher *NHX1* expression in the roots (Fig. 3A). In the roots, higher *NHX3* expression was significantly and positively correlated with higher accumulation of Na^+^ (Fig. 3B).

**Figure 4.**
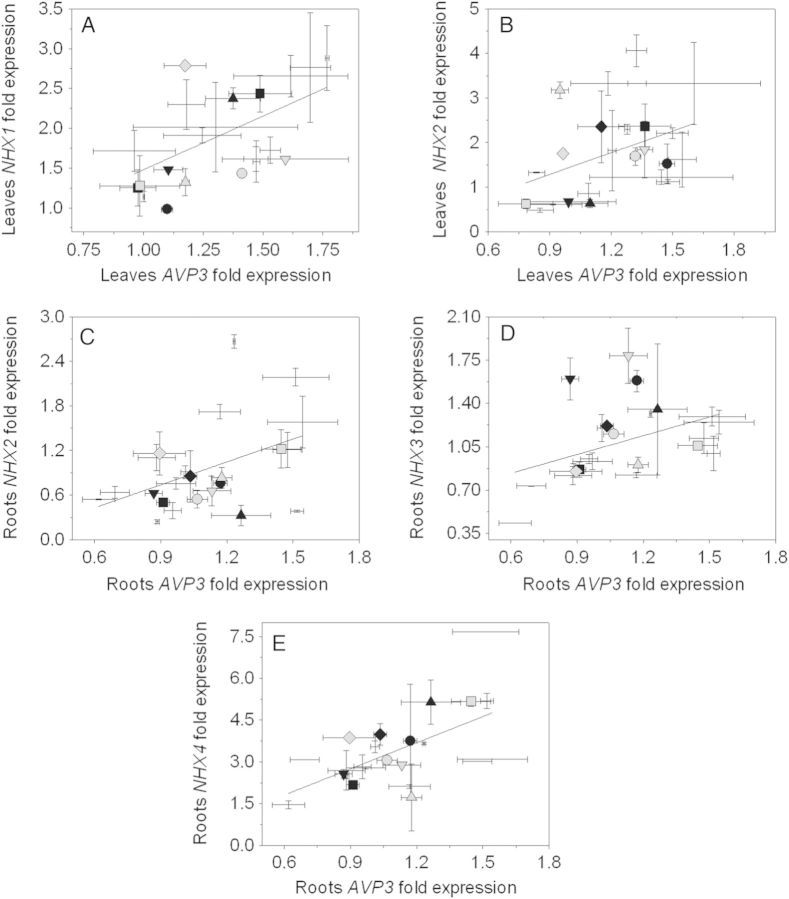
Correlations between expression of (A) *NHX1* and *AVP3* in the leaves; (B) *NHX2* and *AVP3* in the leaves; (C) *NHX2* and *AVP3* in the roots; (D) *NHX3* and *AVP3* in the roots; and (E) *NHX4* and *AVP3* in the roots. Black symbols represent the five accessions with higher PTI values: LA 1325 (square), PI 126443 (rhombus), LA 1522 (up-pointing triangle), LA 1340 (down-pointing triangle) and LA 1245 (filled circle). Grey symbols represent the five accessions with lower PTI values: LA 1938 (square), LA 3320 (rhombus), LA 2711 (up-pointing triangle), LA 2548 (down-pointing triangle) and OT 2209 (filled circle). Values indicate the means ± SE of three biological replicas. Values indicate the means ± SE of three biological replicas. All correlations tested using the Pearson correlation coefficient. All correlations are statistically significant.

The analysis of the gene expression of *NHX* and *AVP* genes showed some statistically significant correlations (Fig. 4). Both *NHX1* (Fig. 4A) and *NHX2* (Fig. 4B) expressions positively correlated with *AVP3* expression in the leaves. In the roots, *NHX2* (Fig. 4C), *NHX3* (Fig. 4D) and *NHX4* (Fig. 4E) expressions were also positively correlated with *AVP3* expression. Interestingly, no clear trend in these correlations was observed regarding accessions with high and low PTI scores.

### Na^+^ accumulation and *HKT*, *SOS* and *AHA* gene expression

**Figure 5.**
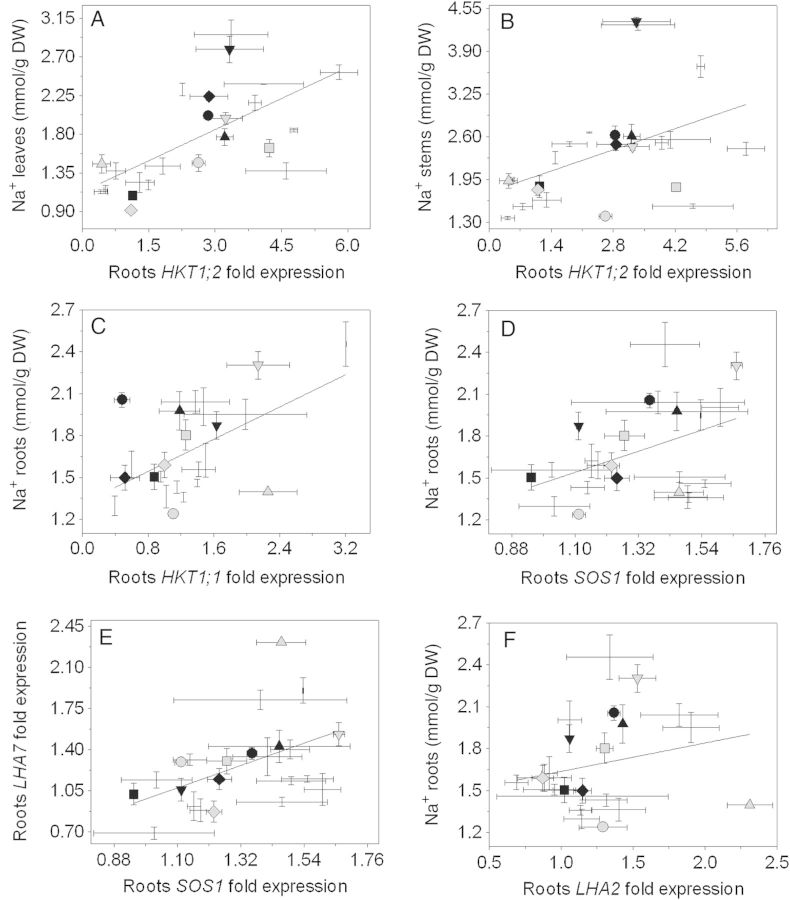
Na^+^ accumulation in the (A) leaves and (B) in the stems and its correlation with *HKT1;2* and (C) *HKT1;1* expression in the roots, (D) Na^+^ accumulation in the roots and its correlation with *SOS1* expression in the roots, (E) *LHA7* and *SOS1* expression in the roots, (F) Na^+^ accumulation and *LHA2* expression in the roots. Black symbols represent the five accessions with higher PTI values: LA 1325 (square), PI 126443 (rhombus), LA 1522 (up-pointing triangle), LA 1340 (down-pointing triangle) and LA 1245 (filled circle). Grey symbols represent the five accessions with lower PTI values: LA 1938 (square), LA 3320 (rhombus), LA 2711 (up-pointing triangle), LA 2548 (down-pointing triangle) and OT 2209 (filled circle). Values indicate the means ± SE of three biological replicas. The Pearson correlation coefficient was used in (C) and (D) and the Spearman correlation coefficient was used in (A), (B) and (E). All correlations are statistically significant.

Na^+^ accumulation correlated with expression of several genes involved in the Na^+^ homoeostasis *in planta* (Fig. 5). Both Na^+^ concentrations measured in leaves (Fig. 5A) and stems (Fig. 5B) correlated with *HKT1;2* expression in roots. From the 23 accessions studied, only LA 2695, LA 2860, Abigail F1 and LA 2711 showed a reduction in the root *HKT1;2* expression when treated with NaCl. In roots, Na^+^ concentration correlated with *HKT1;1* (Fig. 5C), *SOS1* (Fig. 5D) and *AHA2* (Fig. 5F) expression in the roots. Also in the roots, a positive significant correlation between *SOS1* and *AHA7* expressions was observed (Fig. 5E).

### Correlation analysis and PCA

**Figure 6.**
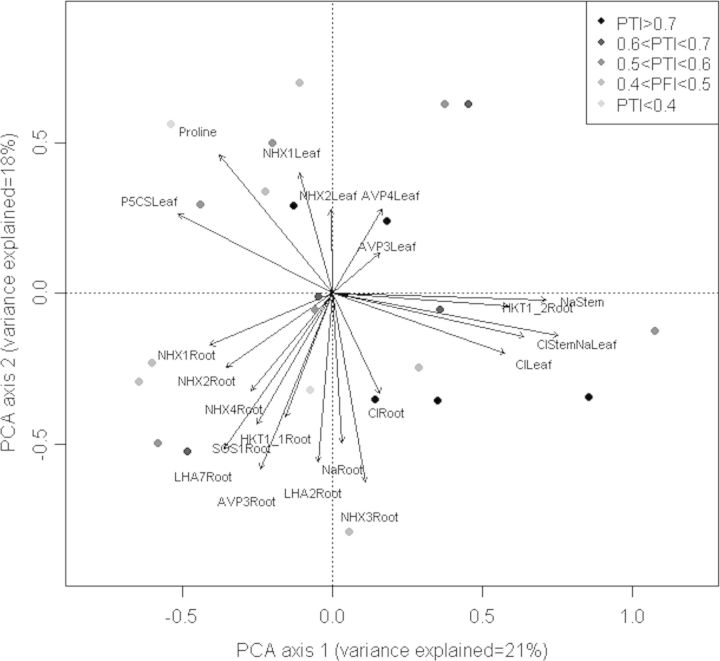
Two axes of a principal component (PC1 and PC2) analysis showing the position of several physiological parameters. Arrows indicate eigenvectors representing the strength and direction of the trait correlation relative to the first two principal components (PC1 and PC2). Dots indicate the PTI values of the accessions analysed in this study.

Due to the great variation in the parameters between the accessions, and because of the statistically significant correlations observed among some of these parameters (Table 1) we decided to perform a principal component analysis (PCA), (Fig. 6). With this analysis we aimed to reveal the contribution of the correlations to the differences in salinity tolerance observed in Table 1.

**Table 1.** Linear correlation coefficients between ion concentrations measured in leaf, stem and root tissue of salt-treated plants; proline concentration measured in the leaves of salt-treated plants; and gene expression of several genes involved in the transport of Na^+^. Ion and proline concentrations used in these correlations were measured only in salt-treated samples. Proline was only measured in leaf tissue. The asterisks show statistically significant correlations, *P*< 0.05.

	Leaves Na^+^	Stems Na^+^	Roots Na^+^	Proline	Roots *AHA7*	Leaves *AVP4*	Leaves *AVP3*	Roots *AVP3*
Leaves Cl^−^	0.419*							
Stems Cl^−^		0.493*						
Roots *HKT1;2*	0.414*	0.188*						
Roots *HKT1;1*			0.308*					
Roots *SOS1*					0.230*			
Roots *AHA2*			0.363*					
Roots *NHX1*	0.1887*							
Roots *NHX3*			0.259*					0.181*
Roots *NHX2*								0.200*
Roots *NHX4*								0.363*
Leaves *NHX1*						0.313*		
Leaves *NHX2*							0.163*	
Leaves *P5CS*	0.271*			0.205*

The patterns of the variables co-variation are shown by PCA, and eigenvectors represent the strength, indicated by the length of the vector, and direction of the trait correlation in relation to the first two principal components (PCs) (Fig. 6). Both PCs accounted for 39 % of the total variance. Both Na^+^ and Cl^−^ accumulation in the stem and in the leaf were explained by the major PC1. In PC1 Na^+^ accumulation in the stems and in the leaves, Cl^−^ accumulation in the stems and in the leaves and *HKT1;2* expression in the roots were strongly associated. Na^+^ accumulation in the roots and *LHA2*, *NHX3* and *AVP3* expressions in the roots were explained by the PC2 as they did not significantly contribute to other PCs.

## Discussion

### Salinity tolerance does not correlate with shoot Na^+^ concentrations

For several crop species salinity tolerance has been linked to an effective Na^+^ exclusion from the shoots (Gorham *et al.* 1990; Schachtman and Munns 1992; Forster 2001; Zhu *et al.* 2001; Munns and James 2003; Garthwaite *et al.* 2005). Accumulation or exclusion of Na^+^ from the shoots under saline conditions is based on the capacity of the plant to reduce the uptake of Na^+^ (Koval and Koval 1996). In tomato, controlled Na^+^ accumulation in the shoot may be an important factor in enhancing salt tolerance (Perez-Alfocea *et al.* 1996; Cuartero and Fernandez-Munoz 1999). Nevertheless, some studies show that the link between Na^+^ exclusion from the shoots and salinity tolerance is not as clear as previously assumed (Rus *et al.* 2006; Genc *et al.* 2010; Jha *et al.* 2010). Similarly, in this study, no correlation was observed between Na^+^ accumulation in any of the tissues analysed and tolerance to salinity. For example, both LA 1325 (tolerant) and LA 3320 (sensitive) showed similar Na^+^ accumulations in the leaves **[see** **Supporting Information****]**. Regarding K^+^, tomato accessions with high and low PTI scores—LA 1325, LA 3320 and OT 2209—were able to maintain constant concentrations of K^+^ in all tissues analysed when treated with NaCl.

In a study with *Arabidopsis* ecotypes, slower growth rates correlated with increased salinity tolerance (Jha *et al.* 2010). In this way, accessions with slower growth in control conditions showed higher salinity tolerance when growing under salinity stress conditions (Jha *et al.* 2010). In this study, we observed this only for some of the accessions. Slow growth means reduced transpiration and reduced water uptake and, consequently, reduced Na^+^ uptake via the roots, which might allow more time and better Na^+^ partitioning in the shoots. This suggests that for the results observed in our study, except Na^+^ exclusion, other mechanisms involved in Na^+^ tissue tolerance might play an important role in salinity tolerance.

### *P5CS* expression is related to Na^+^, proline and sucrose accumulation in the leaves

In this study, a positive correlation between the expression of *P5CS* and the accumulation of proline in the leaves was observed (Fig. 2B). When accessions were analysed individually, it was possible to see that accessions with high and low PTI scores had the same proline accumulation in the leaves. This agrees with studies reporting high accumulation of proline in tolerant (Jain *et al.* 1991; Kirti *et al.* 1991; Juan *et al.* 2005) and sensitive (Tal *et al.* 1979) tomato accessions. It has been suggested that proline accumulation is related to lower concentrations of Na^+^ in the leaves. However, Juan *et al.* (2005) reported that proline accumulation and biomass production are unrelated. The same result was observed in our study. Despite the significant increase in proline accumulation due to the salinity treatment shown by all accessions, no correlation between proline and PTI was observed. This suggests that, although proline accumulation is a common response to salinity stress, it is not, *per se*, the driving force for salinity tolerance in tomato.

There is also some controversy about the role of sugars in salinity tolerance (Juan *et al.* 2005). Despite a reduction in net CO_2_ assimilation during salinity stress (Murakeozy *et al.* 2003; Ashraf and Harris 2004), a higher accumulation of soluble sugars in stressed plants has widely been reported. Nevertheless, in a study by Ashraf (1997), this hypothesis was not confirmed. Juan *et al.* (2005) showed that the highest and lowest accumulations of sucrose were observed in the most and in the least tolerant cultivars, respectively. The same authors reported that Na^+^ and Cl^−^ concentrations, as well as biomass, correlated with the concentration of sucrose (Juan *et al.* 2005). In our study, despite the significant increases in sucrose accumulation observed in all accessions, no correlation between sucrose and PTI or Na^+^ or Cl^−^ was observed.

### Role of *NHXs* and *AVPs* in Na^+^, K^+^ and Cl^−^ homoeostasis

In this study, we observed that high expression in the roots of *NHX1* and *NHX3* correlated with low accumulation of Na^+^ in the leaves and high accumulation of Na^+^ in the roots, respectively (Fig. 3A and B). This agreed with previously published results, since NHX1 is involved in Na^+^ and K^+^ sequestration in the vacuole (Apse *et al.* 1999; Venema *et al.* 2003), and *NHX3* was predominantly expressed in root tissue (Maathuis *et al.* 2003). However, these results were opposed to those obtained by Villalta *et al.* (2008) who map *SlNHX3* to a QTL related to Na^+^ accumulation in the leaves. Although Villalta *et al.* (2007) reported that *SlNHX1* is associated with a QTL for Cl^−^ concentration in young leaves, we did not find a clear correlation between *SlNHX1* expression and Cl^−^ accumulation in the leaves. Interestingly, *SlNHX2* expression did not correlate with K^+^ concentrations in any of the tissues analysed in this study, albeit its ubiquitous expression pattern (Venema *et al.* 2003). *NHXs* expression levels were positively correlated with *AVPs* expression (Fig. 4). Although *AVP* has no direct role in Na^+^ homoeostasis, its ability to create a proton gradient between the vacuole and the cytosol (Munns and Tester 2008) can energize the activity of NHX (Gaxiola *et al.* 2001; Maathuis *et al.* 2003; Jha *et al.* 2010).

### Role of *HKT1;1, HKT1;2, SOS1 and AHA* in Na^+^ homoeostasis

In this study, a positive correlation between root *HKT1;2* expression and the concentrations of Na^+^ measured in the leaves and in the stems, but not in the roots was observed. In the roots, the expression of *HKT1;1* and the accumulation of Na^+^ were correlated. These results can be considered surprising as the tomato *HKT1;2* sequence is more similar to *AtHKT1;1* than tomato *HKT1;1* (Asins *et al.* 2012), and because *AtHKT1;1* expression in the roots is associated with lower Na^+^ accumulation in the shoots (Berthomieu *et al.* 2003; Sunarpi *et al.* 2005; Davenport *et al.* 2007; Moller *et al.* 2009; Jha *et al.* 2010; Plett *et al.* 2010). Interestingly, both accessions with the highest and lowest PTI scores, LA 1245 and OT 2209, respectively, showed similar *HKT1;2* expression in their roots, but different Na^+^ accumulations in their shoots. In the case of *HKT1;1,* both accessions with the highest PTI values showed reduced *HKT1;1* expression in the roots when treated with salt. Nevertheless, these two accessions showed different behaviours in terms of Na^+^ accumulation in the roots; hence, LA 1245 accumulated more Na^+^ than PI 126443. In a work by Rus *et al.* (2006), the correlation between weak *AtHKT1;1* alleles of two *Arabidopsis thaliana* ecotypes and high Na^+^ accumulation in the shoots is described, this is in contrast with the tomato ecotypes from this study, which showed low Na^+^ accumulation. Nevertheless, the results we obtained with *HKT1;1* should be treated with caution. When expressed in *Xenopus laevis* oocytes, SlHKT1;1 did not produce any measurable currents (P.A., unpubl. res.), in contrast with the results reported by Asins *et al.* (2012). This group reported that, when expressed in yeast cells, SlHKT1;1, but not SlHKT1;2, depleted the Na^+^ present in the growing medium (Asins *et al.* 2012). This can result from a deficient translation of the protein, when expressed in heterologous systems (Haro *et al.* 2005).

In this study, high *SOS1* expression correlated with high Na^+^ accumulation in the roots. This might be explained by the need of stressed plants to pump more Na^+^ out of the cell, either via loading of Na^+^ into the xylem or via extrusion of Na^+^ back to the growth medium. Here, we could observe a positive correlation between *SOS1* and *AHA7* expression as well as a positive correlation between *AHA2* and Na^+^ accumulation, both in the roots. This suggests that *SOS1* is energized by *AHA7*, but not *AHA2*. This is interesting, as Maathuis *et al.* (2003) reported that the energization of Na^+^/H^+^ extrusion at the root soil boundary is likely to be driven by *AHA2*. Nevertheless, AHA2 is also involved in the Na^+^ homoeostasis in the root, possibly via an indirect mechanism, shown by the positive correlation between *AHA2* and Na^+^ accumulation.

## Conclusions

In conclusion, several correlations were observed among the different genes analysed, and between different genes and ions or proline concentrations. For instance, Na^+^ concentrations in both the leaves and stems were positively correlated with *HKT1;2* expression in the roots, and Na^+^ concentration measured in the roots was positively correlated with *HKT1;1* expression also in the roots. Higher and lower Na^+^ accumulation in the roots and leaves were significantly correlated with higher *NHX3* and *NHX1* expression in the roots, respectively. However, accessions with high and low PTI scores had similar concentrations of ions, proline and sucrose and gene expression levels, showing that the maintenance of growth in the presence of 100 mM NaCl did not correlate with the exclusion or accumulation of Na^+^. This suggests that in tomato, for a particular level of salinity tolerance, a complex ratio between Na^+^ exclusion and tissue tolerance defines the salinity tolerance of individual tomato accessions. In tomato it is likely that the mechanisms of tissue and salinity tolerance work independently, making salinity tolerance depend on their relative effects rather than on one of these mechanisms alone.

## Sources of Funding

This work was carried out within the PhD project of P.A. ‘Mayas: making *Solanum lycopersicum* more salt tolerant’ and was supported by Enza Zaden, Enkhuizen, the Netherlands.

## Contributions by the Authors

P.A., A.H.d.B. and G.-J.d.B. conceived and designed the experiments. P.A. and R.F. performed the experiments. P.A. analysed the data. P.A. and A.H.d.B. wrote the paper.

## Conflicts of Interest Statement

None declared.

## Supporting Information

The following Supporting Information is available in the online version of this article:

**File 1. Table**. Primers used in the qRT–PCR studies.

**File 2. Table**. Tolerance to salinity of 23 tomato accessions grown in rockwool plugs soaked with Hoagland's solution and treated with either 0 or 100 mM NaCl for 2 weeks. Values indicate the means ± SE of three biological replicates. The asterisks indicate significant differences according to Student's *t*-test (*P < 0.05).

**File 3. Table**. List of Na^+^, K^+^, Cl^−^ and Na^+^/K^+^ and concentrations in the (A) roots, (B) stems and (C) leaves of 23 tomato accessions grown in rockwool plugs soaked with Hoagland's solution for 3 weeks before treated to either 0 or 100 mM NaCl for 2 weeks. Values indicate the means ± SE of three biological replicas. The asterisks indicate significant differences according to one-way ANOVA (*P < 0.05).

Additional Information
