# Supplementary material for: Role of Na+, K+, Cl−, proline and sucrose concentrations in determining salinity tolerance and their correlation with the expression of multiple genes in tomato
Source: AoB Plants. 2014 Jul 4;6:plu039. doi: 10.1093/aobpla/plu039 (PMC4122256; doi:10.1093/aobpla/plu039)
Supplement: Additional Information [file supp_6_plu039_index.html]

Role of Na+, K+, Cl−, proline and sucrose concentrations in determining salinity tolerance and their correlation with the expression of multiple genes in tomato — Additional Information 

# Role of Na+, K+, Cl−, proline and sucrose concentrations in determining salinity tolerance and their correlation with the expression of multiple genes in tomato

## Additional Information

Additional Information

**Files in this Data Supplement:**

- Additional Information Table 1 - docx file
- Additional Information Table 2 - docx file
- Additional Information Table 3 - docx file
