# Supplementary material for: Role of Na+, K+, Cl−, proline and sucrose concentrations in determining salinity tolerance and their correlation with the expression of multiple genes in tomato
Source: AoB Plants. 2014 Jul 4;6:plu039. doi: 10.1093/aobpla/plu039 (PMC4122256; doi:10.1093/aobpla/plu039)
Supplement: Additional Information [file supp_plu039_plu039supp_table1.docx]

| **Primer Name** | **Primer Sequence (from 5' to 3')** | **Reference** |
| --- | --- | --- |
| *SlAHA2 F* | AGAGAAGAGCAGAGATGGCA | This study |
| *SlAHA2 R* | ACCTTTCAGCTTCACCACTGATT | This study |
| *SlAHA7 F* | CTTGGTGGCTACTTGGCAAT | This study |
| *SlAHA7 R* | GATAGTGCTTACTTGAAGGT | This study |
| *SlHKT1;1 F* | ACTAGCCCAAGAAACTCAAAT | (Asins et al. 2012) |
| *SlHKT1;1 R* | CTAATGTTACAACTCCAAGGAATT | (Asins et al. 2012) |
| *SlHKT1;2 F* | TGAGCTAGGGAATGTAATAAACG | (Asins et al. 2012) |
| *SlHKT1;2 R* | AGAGAGAAACTAACGATGAACC | (Asins et al. 2012) |
| *SlSOS1 F* | TCGAGTGATGATTCTGGTGG | (Huertas et al. 2012) |
| *SlSOS1 R* | GAGCCTTTCCACACTGTGAT | (Huertas et al. 2012) |
| *SlNHX1 F* | GACAGTCCTGGAAAATCT | (Galvez et al. 2012) |
| *SlNHX1 R* | GGTTATCAGCCCAAACACC | (Galvez et al. 2012) |
| *SlNHX2 F* | CCTTTGAGGGGAACAA TGG | (Huertas et al. 2012) |
| *SlNHX2 R* | CA TCTTCA TCTTCGTCTCC | (Huertas et al. 2012) |
| *SlNHX3 F* | CTCAAGAGTCACCACCAAGCA | (Galvez et al. 2012) |
| *SlNHX3 R* | CCAACCAAAACAAGACCCAACA | (Galvez et al. 2012) |
| *SlNHX4 F* | TGTGGTGGCAGCAGGAGACTTA | (Huertas et al. 2012) |
| *SlNHX4 R* | TGGTGGGCAGGTTTGA TGAGAG | (Huertas et al. 2012) |
| *SlAVP3 F* | TGGAATGAGCCACAGAATCA | This study |
| *SlAVP3 R* | ATTGCACCGACTAGCAGACC | This study |
| *SlAVP4 F* | GCTACAACCAAGGGATTTGC | This study |
| *SlAVP4 R* | AGTTCGACCAACAGCTGCAC | This study |
| *SlP5CS F* | TCGAGCATTTGTGAAGAACG | This study |
| *SlP5CS R* | CAGCACCAGAAGTCACCAAA | This study |
| *SlEF1α F* | GACAGGCGTTCAGGTAAGGA | (Huertas et al. 2012) |
| *SlEF1α R* | GGGTATTCAGCAAAGGTCTC | (Huertas et al. 2012) |
