# Supplementary material for: Role of Na+, K+, Cl−, proline and sucrose concentrations in determining salinity tolerance and their correlation with the expression of multiple genes in tomato
Source: AoB Plants. 2014 Jul 4;6:plu039. doi: 10.1093/aobpla/plu039 (PMC4122256; doi:10.1093/aobpla/plu039)
Supplement: Additional Information [file supp_plu039_plu039supp_table2.docx]

| Accession | Species/ Name | | | Treatment  (NaCl) | Shoot Fresh  Mass (g) | PTI  (FWshoots salt/ FWshoots control) |
| --- | --- | --- | --- | --- | --- | --- |
| Arbasson F1 | *Solanum lycopersicum* | | | 0 mM | 5.24±1.38 * | 0.46±0.06 |
|  |  |  |  | 100 mM | 2.36±0.54 |  |
| LA 1938 | *Solanum chilense* | | | 0 mM | 2.47±0.87 * | 0.37±0.05 |
|  |  |  |  | 100 mM | 0.89±0.25 |  |
| LA 1959 | *Solanum chilense* | | | 0 mM | 2.00±0.13 * | 0.48±0.08 |
|  |  |  |  | 100 mM | 0.97±0.29 |  |
| LA 1325 | *Solanum chmielewskii* | | | 0 mM | 0.59±0.01 | 0.71±0.14 |
|  |  |  |  | 100 mM | 0.42±0.14 |  |
| LA 2695 | *Solanum chmielewskii* | | | 0 mM | 1.10±0.10 * | 0.53±0.06 |
|  |  |  |  | 100 mM | 0.59±0.16 |  |
| GI 568 | *Solanum corneliomuelleri* | | | 0 mM | 2.31±0.60 * | 0.66±0.12 |
|  |  |  |  | 100 mM | 1.44±0.21 |  |
| PI 126443 | *Solanum corneliomuelleri* | | | 0 mM | 1.78±0.21 | 0.71±0.13 |
|  |  |  |  | 100 mM | 1.23±0.26 |  |
| LA 0532 | *Solanum galapagense* | | | 0 mM | 1.30±0.16 * | 0.50±0.06 |
|  |  |  |  | 100 mM | 0.65±0.09 |  |
| LA 0317 | *Solanum galapagense* | | | 0 mM | 2.77±0.14 * | 0.50±0.06 |
|  |  |  |  | 100 mM | 1.37±0.23 |  |
| G 1560 | *Solanum habrochaites* | | | 0 mM | 2.81±0.28 * | 0.60±0.09 |
|  |  |  |  | 100 mM | 1.67±0.25 |  |
| LA 2167 | *Solanum habrochaites* | | | 0 mM | 1.34±0.16 * | 0.67±0.08 |
|  |  |  |  | 100 mM | 0.89±0.13 |  |
| LA 2860 | *Solanum habrochaites glabratum* | | | 0 mM | 2.85±0.27 * | 0.56±0.05 |
|  |  |  |  | 100 mM | 1.60±0.31 |  |
| PI 126449 | *Solanum habrochaites glabratum* | | | 0 mM | 2.20±0.21 * | 0.65±0.08 |
|  |  |  |  | 100 mM | 1.41±0.21 |  |
| LA 3320 | *Solanum lycopersicum* | | | 0 mM | 6.65±1.10 * | 0.39±0.08 |
|  |  |  |  | 100 mM | 2.52±0.73 |  |
| Abigail F1 | *Solanum lycopersicum* | | | 0 mM | 4.17±1.84 | 0.57±0.13 |
|  |  |  |  | 100 mM | 1.71±0.03 |  |
| LA 2711 | *Solanum lycopersicum* | | | 0 mM | 5.33±0.33 * | 0.43±0.07 |
|  |  |  |  | 100 mM | 2.27±0.55 |  |
| LA 2194 | *Solanum neorickii* | | | 0 mM | 1.60±0.42 * | 0.54±0.11 |
|  |  |  |  | 100 mM | 0.82±0.05 |  |
| LA 1340 | *Solanum pennellii* | | | 0 mM | 0.87±0.08 | 0.77±0.12 |
|  |  |  |  | 100 mM | 0.65±0.11 |  |
| LA 1522 | *Solanum pennellii* | | | 0 mM | 3.32±0.49 | 0.76±0.12 |
|  |  |  |  | 100 mM | 2.46±0.30 |  |
| LA 1302 | *Solanum pennellii puberulum* | | | 0 mM | 1.78±0.29 * | 0.57±0.16 |
|  |  |  |  | 100 mM | 0.96±0.29 |  |
| LA 2548 | *Solanum peruvianum* | | | 0 mM | 2.09±0.54 * | 0.44±0.04 |
|  |  |  |  | 100 mM | 0.90±0.11 |  |
| OT 2209 | *Solanum pimpinellifolium* | | | 0 mM | 4.31±0.24 * | 0.44±0.05 |
|  |  |  |  | 100 mM | 1.92±0.39 |  |
| LA 1245 | *Solanum pimpinellifolium* | | | 0 mM | 1.26±0.22 | 0.98±0.11 |
|  |  |  |  | 100 mM | 1.25±0.44 |  |
