# Supplementary material for: Role of Na+, K+, Cl−, proline and sucrose concentrations in determining salinity tolerance and their correlation with the expression of multiple genes in tomato
Source: AoB Plants. 2014 Jul 4;6:plu039. doi: 10.1093/aobpla/plu039 (PMC4122256; doi:10.1093/aobpla/plu039)
Supplement: Additional Information [file supp_plu039_plu039supp_table3.docx]

**A)**

| **Accession** | **Species** | PTI  (FWshoots salt/ FWshoots control) | Treat  ment  mM  NaCl | Roots  [Na^+^] | Roots  [K^+^] | Roots  Na^+^/K^+^ | Roots  [Cl^-^] |
| --- | --- | --- | --- | --- | --- | --- | --- |
| Arbasson F1 | *S. lycopersicum* | 0.46±0.06 | 0 | 0.50±0.01 | 1.44±0.01 | 0.35±0.01 | 0.39±0.03 |
|  |  |  | 100 | 1.46±0.03* | 1.06±0.01* | 1.38±0.02* | 1.57±0.01* |
| LA 1938 | *S. chilense* | 0.37±0.05 | 0 | 0.25±0.03 | 1.16±0.08 | 0.22±0.03 | 0.17±0.00 |
|  |  |  | 100 | 1.80±0.11* | 1.12±0.04 | 1.60±0.07* | 1.32±0.17* |
| LA 1959 | *S. chilense* | 0.48±0.08 | 0 | 0.48±0.02 | 0.87±0.04 | 0.56±0.02 | 0.28±0.01 |
|  |  |  | 100 | 1.43±0.05* | 0.72±0.04 | 2.00±0.11* | 1.75±0.11* |
| LA 1325 | *S. chmielewskii* | 0.71±0.14 | 0 | 0.55±0.02 | 1.12±0.03 | 0.49±0.02 | 0.28±0.02 |
|  |  |  | 100 | 1.50±0.09* | 1.00±0.05 | 1.51±0.09* | 0.80±0.05* |
| LA 2695 | *S. chmielewskii* | 0.53±0.06 | 0 | 0.66±0.04 | 1.06±0.03 | 0.62±0.05 | 0.76±0.07 |
|  |  |  | 100 | 1.56±0.05* | 1.41±0.10* | 1.11±0.06* | 1.20±0.13* |
| GI 568 | *S. corneliomuelleri* | 0.66±0.12 | 0 | 0.29±0.02 | 1.08±0.03 | 0.27±0.03 | 0.30±0.01 |
|  |  |  | 100 | 1.36±0.04* | 0.71±0.05* | 1.93±0.17* | 1.12±0.12* |
| PI 126443 | *S. corneliomuelleri* | 0.71±0.13 | 0 | 0.42±0.01 | 1.20±0.04 | 0.36±0.01 | 0.43±0.00 |
|  |  |  | 100 | 1.50±0.09* | 1.35±0.07 | 1.11±0.07* | 1.95±0.30* |
| LA 0532 | *S. galapagense* | 0.50±0.06 | 0 | 0.38±0.02 | 1.23±0.04 | 0.31±0.02 | 0.13±0.03 |
|  |  |  | 100 | 1.30±0.07* | 1.96±0.11* | 0.67±0.06* | 1.13±0.01* |
| LA 0317 | *S. galapagense* | 0.50±0.06 | 0 | 0.36±0.02 | 1.11±0.01 | 0.33±0.02 | 0.18±0.01 |
|  |  |  | 100 | 1.59±0.10* | 1.26±0.12 | 1.28±0.09* | 0.78±0.11* |
| G 1560 | *S. habrochaites* | 0.60±0.09 | 0 | 0.39±0.04 | 1.53±0.02 | 0.25±0.03 | 0.33±0.04 |
|  |  |  | 100 | 1.51±0.04* | 0.84±0.06* | 1.82±0.12* | 1.42±0.08* |
| LA 2167 | *S. habrochaites* | 0.67±0.08 | 0 | 0.55±0.02 | 0.94±0.03 | 0.58±0.01 | 0.18±0.01 |
|  |  |  | 100 | 2.04±0.08* | 0.67±0.04* | 3.05±0.21* | 1.71±0.01* |
| LA 2860 | *S. habrochaites glabratum* | 0.56±0.05 | 0 | 0.44±0.00 | 1.06±0.02 | 0.42±0.00 | 0.28±0.00 |
|  |  |  | 100 | 1.95±0.11* | 0.97±0.05 | 2.02±0.19* | 1.09±0.02* |
| PI 126449 | *S. habrochaites glabratum* | 0.65±0.08 | 0 | 0.37±0.01 | 1.12±0.04 | 0.33±0.01 | 0.15±0.02 |
|  |  |  | 100 | 2.46±0.16* | 1.48±0.10* | 1.68±0.15* | 0.81±0.09* |
| LA 3320 | *S. lycopersicum* | 0.39±0.08 | 0 | 0.68±0.02 | 1.13±0.03 | 0.60±0.00 | 0.30±0.01 |
|  |  |  | 100 | 1.59±0.09* | 1.40±0.18 | 1.16±0.10* | 1.27±0.04* |
| Abigail F1 | *S. lycopersicum* | 0.57±0.13 | 0 | 0.39±0.04 | 1.13±0.04 | 0.34±0.04 | 0.45±0.00 |
|  |  |  | 100 | 1.36±0.08* | 1.23±0.06 | 1.11±0.02* | 1.17±0.10* |
| LA 2711 | *S. lycopersicum* | 0.43±0.07 | 0 | 0.58±0.05 | 1.28±0.04 | 0.46±0.05 | 0.35±0.02 |
|  |  |  | 100 | 1.40±0.01* | 1.49±0.09 | 0.94±0.06* | 1.32±0.06* |
| LA 2194 | *S. neorickii* | 0.54±0.11 | 0 | 0.49±0.05 | 0.87±0.06 | 0.56±0.02 | 0.40±0.06 |
|  |  |  | 100 | 2.00±0.14* | 0.97±0.06 | 2.08±0.12* | 0.95±0.10* |
| LA 1340 | *S. pennellii* | 0.77±0.12 | 0 | 0.23±0.02 | 1.20±0.03 | 0.19±0.02 | 0.24±0.04 |
|  |  |  | 100 | 1.87±0.10* | 1.50±0.11 | 1.25±0.05* | 1.37±0.13* |
| LA 1522 | *S. pennellii* | 0.76±0.12 | 0 | 0.44±0.02 | 1.35±0.05 | 0.33±0.02 | 0.21±0.02 |
|  |  |  | 100 | 1.96±0.14* | 1.56±0.12 | 1.27±0.07* | 0.98±0.05* |
| LA 1302 | *S. pennellii puberulum* | 0.57±0.16 | 0 | 0.27±0.01 | 1.54±0.04 | 0.18±0.01 | 0.50±0.04 |
|  |  |  | 100 | 1.62±0.12* | 1.49±0.08 | 1.08±0.03* | 1.94±0.27* |
| LA 2548 | *S. peruvianum* | 0.44±0.04 | 0 | 0.59±0.04 | 1.54±0.08 | 0.38±0.01 | 0.29±0.04 |
|  |  |  | 100 | 2.30±0.10* | 1.49±0.08 | 1.55±0.02* | 2.47±0.20* |
| OT 2209 | *S. pimpinellifolium* | 0.44±0.05 | 0 | 0.44±0.02 | 0.87±0.02 | 0.50±0.02 | 0.38±0.03 |
|  |  |  | 100 | 1.24±0.03* | 0.71±0.09 | 1.79±0.23* | 1.07±0.01* |
| LA 1245 | *S. pimpinellifolium* | 0.98±0.11 | 0 | 0.32±0.02 | 1.15±0.04 | 0.28±0.03 | 0.40±0.01 |
|  |  |  | 100 | 2.05±0.05* | 1.68±0.10* | 1.24±0.10* | 0.80±0.01* |

**B)**

| **Accession** | **Species** | PTI  (FWshoots salt/ FWshoots control) | Treat  ment  mM  NaCl | Stems  [Na^+^] | Stems  [K^+^] | Stems  Na^+^/K^+^ | Stems  [Cl^-^] |
| --- | --- | --- | --- | --- | --- | --- | --- |
| Arbasson F1 | *S. lycopersicum* | 0,46±0,06 | 0 | 0,92±0,02 | 2,72±0,01 | 0,34±0,01 | 0,52±0,02 |
|  |  |  | 100 | 2,28±0,10* | 2,10±0,08 | 1,08±0,03* | 1,77±0,00* |
| LA 1938 | *S. chilense* | 0,37±0,05 | 0 | 1,06±0,03 | 2,37±0,11 | 0,45±0,03 | 0,35±0,02 |
|  |  |  | 100 | 1,83±0,03* | 1,90±0,07* | 0,97±0,03* | 1,96±0,04* |
| LA 1959 | *S. chilense* | 0,48±0,08 | 0 | 1,06±0,01 | 2,03±0,05 | 0,52±0,02 | 0,55±0,00 |
|  |  |  | 100 | 2,51±0,10* | 1,49±0,07* | 1,68±0,04* | 2,56±0,13* |
| LA 1325 | *S. chmielewskii* | 0,71±0,14 | 0 | 0,60±0,02 | 1,94±0,08 | 0,31±0,01 | 0,25±0,04 |
|  |  |  | 100 | 1,84±0,17* | 1,86±0,06 | 0,99±0,06* | 1,48±0,06* |
| LA 2695 | *S. chmielewskii* | 0,53±0,06 | 0 | 0,50±0,05 | 2,81±0,04 | 0,18±0,01 | 0,13±0,02 |
|  |  |  | 100 | 1,95±0,02* | 2,87±0,12 | 0,68±0,03* | 2,04±0,08* |
| GI 568 | *S. corneliomuelleri* | 0,66±0,12 | 0 | 1,19±0,06 | 2,12±0,04 | 0,56±0,02 | 0,34±0,01 |
|  |  |  | 100 | 2,42±0,10* | 1,25±0,04* | 1,94±0,04* | 2,31±0,01* |
| PI 126443 | *S. corneliomuelleri* | 0,71±0,13 | 0 | 0,96±0,01 | 1,64±0,03 | 0,58±0,01 | 0,41±0,04 |
|  |  |  | 100 | 2,48±0,09* | 1,44±0,06* | 1,73±0,04* | 1,77±0,05* |
| LA 0532 | *S. galapagense* | 0,50±0,06 | 0 | 1,30±0,09 | 2,67±0,12 | 0,49±0,05 | 0,18±0,00 |
|  |  |  | 100 | 3,67±0,17* | 1,74±0,08* | 2,11±0,15* | 1,92±0,02* |
| LA 0317 | *S. galapagense* | 0,50±0,06 | 0 | 1,17±0,05 | 2,40±0,09 | 0,49±0,00 | 0,60±0,07 |
|  |  |  | 100 | 2,49±0,04* | 2,16±0,05 | 1,15±0,04* | 2,23±0,07* |
| G 1560 | *S. habrochaites* | 0,60±0,09 | 0 | 1,02±0,02 | 2,94±0,12 | 0,35±0,01 | 0,51±0,05 |
|  |  |  | 100 | 2,55±0,13* | 1,86±0,02* | 1,37±0,07* | 2,07±0,04* |
| LA 2167 | *S. habrochaites* | 0,67±0,08 | 0 | 0,61±0,05 | 2,46±0,08 | 0,25±0,02 | 0,42±0,01 |
|  |  |  | 100 | 1,64±0,11* | 1,46±0,09* | 1,12±0,03* | 1,66±0,21* |
| LA 2860 | *S. habrochaites glabratum* | 0,56±0,05 | 0 | 0,40±0,00 | 2,54±0,04 | 0,16±0,00 | 0,30±0,02 |
|  |  |  | 100 | 1,36±0,02* | 2,19±0,06* | 0,62±0,01* | 1,90±0,09* |
| PI 126449 | *S. habrochaites glabratum* | 0,65±0,08 | 0 | 0,83±0,00 | 2,21±0,04 | 0,37±0,00 | 0,32±0,03 |
|  |  |  | 100 | 2,66±0,01* | 2,35±0,07 | 1,14±0,03* | 1,60±0,12* |
| LA 3320 | *S. lycopersicum* | 0,39±0,08 | 0 | 0,78±0,03 | 2,47±0,07 | 0,32±0,01 | 0,47±0,01 |
|  |  |  | 100 | 1,79±0,09* | 2,36±0,03 | 0,76±0,04* | 1,49±0,04* |
| Abigail F1 | *S. lycopersicum* | 0,57±0,13 | 0 | 0,78±0,04 | 1,70±0,02 | 0,46±0,03 | 0,57±0,07 |
|  |  |  | 100 | 1,54±0,05* | 1,48±0,15 | 1,06±0,11* | 1,49±0,12* |
| LA 2711 | *S. lycopersicum* | 0,43±0,07 | 0 | 1,11±0,06 | 2,30±0,05 | 0,48±0,04 | 0,47±0,02 |
|  |  |  | 100 | 1,93±0,11* | 1,94±0,04* | 1,00±0,06* | 1,19±0,00* |
| LA 2194 | *S. neorickii* | 0,54±0,11 | 0 | 0,56±0,03 | 2,95±0,05 | 0,19±0,01 | 0,38±0,02 |
|  |  |  | 100 | 1,54±0,03* | 2,38±0,10* | 0,65±0,03* | 2,03±0,06* |
| LA 1340 | *S. pennellii* | 0,77±0,12 | 0 | 1,43±0,07 | 2,79±0,03 | 0,51±0,03 | 0,58±0,02 |
|  |  |  | 100 | 4,35±0,06* | 2,54±0,08* | 1,72±0,03* | 2,72±0,26* |
| LA 1522 | *S. pennellii* | 0,76±0,12 | 0 | 1,56±0,04 | 2,38±0,05 | 0,65±0,02 | 0,22±0,08 |
|  |  |  | 100 | 2,61±0,18* | 1,69±0,10* | 1,56±0,20* | 2,69±0,14* |
| LA 1302 | *S. pennellii puberulum* | 0,57±0,16 | 0 | 1,64±0,08 | 2,85±0,04 | 0,57±0,02 | 0,53±0,03 |
|  |  |  | 100 | 4,30±0,08* | 2,01±0,03* | 2,14±0,05* | 5,11±0,70* |
| LA 2548 | *S. peruvianum* | 0,44±0,04 | 0 | 0,83±0,05 | 2,29±0,02 | 0,36±0,02 | 0,29±0,02 |
|  |  |  | 100 | 2,45±0,05* | 1,59±0,02* | 1,54±0,03* | 1,97±0,05* |
| OT 2209 | *S. pimpinellifolium* | 0,44±0,05 | 0 | 0,50±0,06 | 1,69±0,14 | 0,30±0,01 | 0,33±0,00 |
|  |  |  | 100 | 1,39±0,06* | 1,76±0,08 | 0,79±0,06* | 1,33±0,10* |
| LA 1245 | *S. pimpinellifolium* | 0,98±0,11 | 0 | 1,61±0,02 | 1,77±0,09 | 0,92±0,04 | 0,55±0,01 |
|  |  |  | 100 | 2,62±0,14* | 1,43±0,05* | 1,84±0,08* | 2,08±0,09* |

**C)**

| **Accession** | **Species** | PTI  (FWshoots salt/ FWshoots control) | Treat  ment  mM  NaCl | Leaves  [Na^+^] | Leaves  [K^+^] | Leaves  Na^+^/K^+^ | Leaves  [Cl^-^] |
| --- | --- | --- | --- | --- | --- | --- | --- |
| Arbasson F1 | *S. lycopersicum* | 0,46±0,06 | 0 | 0,36±0,02 | 1,50±0,06 | 0,24±0,00 | 0,23±0,01 |
|  |  |  | 100 | 1,21±0,06* | 1,34±0,02* | 0,90±0,03* | 0,66±0,06* |
| LA 1938 | *S. chilense* | 0,37±0,05 | 0 | 0,34±0,03 | 1,62±0,06 | 0,21±0,01 | 0,26±0,04 |
|  |  |  | 100 | 1,64±0,10* | 1,31±0,04* | 1,26±0,11* | 0,88±0,01* |
| LA 1959 | *S. chilense* | 0,48±0,08 | 0 | 0,36±0,05 | 1,37±0,01 | 0,26±0,03 | 0,16±0,00 |
|  |  |  | 100 | 2,16±0,09* | 1,13±0,03* | 1,91±0,07* | 1,66±0,06* |
| LA 1325 | *S. chmielewskii* | 0,71±0,14 | 0 | 0,22±0,00 | 1,48±0,02 | 0,15±0,00 | 0,09±0,01 |
|  |  |  | 100 | 1,09±0,03* | 1,53±0,10 | 0,72±0,05* | 1,08±0,02* |
| LA 2695 | *S. chmielewskii* | 0,53±0,06 | 0 | 0,22±0,00 | 1,13±0,01 | 0,20±0,00 | 0,24±0,00 |
|  |  |  | 100 | 1,15±0,05* | 1,22±0,02* | 0,94±0,06* | 0,67±0,10* |
| GI 568 | *S. corneliomuelleri* | 0,66±0,12 | 0 | 0,54±0,03 | 1,39±0,10 | 0,39±0,02 | 0,22±0,01 |
|  |  |  | 100 | 2,52±0,09* | 1,27±0,04 | 1,98±0,04* | 1,38±0,01* |
| PI 126443 | *S. corneliomuelleri* | 0,71±0,13 | 0 | 0,60±0,02 | 1,29±0,02 | 0,47±0,02 | 0,23±0,04 |
|  |  |  | 100 | 2,24±0,04* | 1,22±0,04 | 1,83±0,05* | 1,62±0,03* |
| LA 0532 | *S. galapagense* | 0,50±0,06 | 0 | 0,41±0,05 | 1,36±0,06 | 0,31±0,05 | 0,16±0,02 |
|  |  |  | 100 | 1,85±0,02* | 0,93±0,07* | 2,01±0,16* | 1,07±0,00* |
| LA 0317 | *S. galapagense* | 0,50±0,06 | 0 | 0,45±0,07 | 1,43±0,00 | 0,32±0,05 | 0,17±0,00 |
|  |  |  | 100 | 1,43±0,09* | 1,22±0,07* | 1,17±0,07* | 1,03±0,04* |
| G 1560 | *S. habrochaites* | 0,60±0,09 | 0 | 0,83±0,03 | 1,25±0,03 | 0,66±0,03 | 0,09±0,00 |
|  |  |  | 100 | 2,38±0,00* | 0,90±0,03* | 2,65±0,09* | 0,96±0,05* |
| LA 2167 | *S. habrochaites* | 0,67±0,08 | 0 | 0,35±0,02 | 1,60±0,03 | 0,22±0,02 | 0,18±0,01 |
|  |  |  | 100 | 1,24±0,12* | 1,23±0,04* | 1,02±0,13* | 0,75±0,02* |
| LA 2860 | *S. habrochaites glabratum* | 0,56±0,05 | 0 | 0,23±0,02 | 1,32±0,05 | 0,18±0,01 | 0,26±0,03 |
|  |  |  | 100 | 1,13±0,02* | 1,04±0,05* | 1,10±0,05* | 1,17±0,01* |
| PI 126449 | *S. habrochaites glabratum* | 0,65±0,08 | 0 | 0,72±0,02 | 1,28±0,07 | 0,57±0,04 | 0,21±0,00 |
|  |  |  | 100 | 2,32±0,07* | 0,93±0,01* | 2,50±0,05* | 1,35±0,03* |
| LA 3320 | *S. lycopersicum* | 0,39±0,08 | 0 | 0,35±0,03 | 1,53±0,04 | 0,23±0,02 | 0,19±0,02 |
|  |  |  | 100 | 0,91±0,02* | 1,49±0,06 | 0,62±0,02* | 0,84±0,02* |
| Abigail F1 | *S. lycopersicum* | 0,57±0,13 | 0 | 0,30±0,02 | 1,39±0,04 | 0,21±0,02 | 0,11±0,01 |
|  |  |  | 100 | 1,37±0,10* | 1,55±0,03* | 0,88±0,04* | 0,55±0,01* |
| LA 2711 | *S. lycopersicum* | 0,43±0,07 | 0 | 0,30±0,02 | 1,61±0,05 | 0,19±0,02 | 0,25±0,01 |
|  |  |  | 100 | 1,45±0,11* | 1,46±0,03 | 1,00±0,08* | 1,02±0,00* |
| LA 2194 | *S. neorickii* | 0,54±0,11 | 0 | 0,33±0,01 | 1,43±0,05 | 0,23±0,01 | 0,21±0,05 |
|  |  |  | 100 | 1,37±0,10* | 1,44±0,05 | 0,95±0,04* | 1,12±0,00* |
| LA 1340 | *S. pennellii* | 0,77±0,12 | 0 | 0,52±0,05 | 1,55±0,07 | 0,33±0,02 | 0,26±0,03 |
|  |  |  | 100 | 2,79±0,15* | 1,27±0,02* | 2,20±0,11* | 1,75±0,14* |
| LA 1522 | *S. pennellii* | 0,76±0,12 | 0 | 0,62±0,04 | 1,31±0,07 | 0,47±0,02 | 0,27±0,02 |
|  |  |  | 100 | 1,77±0,10* | 1,13±0,03 | 1,56±0,09* | 1,51±0,08* |
| LA 1302 | *S. pennellii puberulum* | 0,57±0,16 | 0 | 0,58±0,03 | 1,35±0,02 | 0,43±0,02 | 0,10±0,00 |
|  |  |  | 100 | 2,96±0,17* | 1,33±0,07 | 2,23±0,11* | 1,77±0,06* |
| LA 2548 | *S. peruvianum* | 0,44±0,04 | 0 | 0,53±0,03 | 1,22±0,01 | 0,43±0,02 | 0,17±0,01 |
|  |  |  | 100 | 1,98±0,07* | 0,92±0,03* | 2,16±0,03* | 0,69±0,04* |
| OT 2209 | *S. pimpinellifolium* | 0,44±0,05 | 0 | 0,30±0,02 | 1,36±0,05 | 0,22±0,01 | 0,12±0,03 |
|  |  |  | 100 | 1,46±0,10* | 1,31±0,03 | 1,11±0,07* | 0,90±0,05* |
| LA 1245 | *S. pimpinellifolium* | 0,98±0,11 | 0 | 0,45±0,03 | 1,21±0,03 | 0,37±0,02 | 0,29±0,03 |
|  |  |  | 100 | 2,02±0,02* | 1,22±0,04 | 1,65±0,05* | 2,30±0,00* |
